# A new genus and species of Thyasiridae (Mollusca, Bivalvia) from deep-water, Beaufort Sea, northern Alaska

**DOI:** 10.3897/zookeys.462.6790

**Published:** 2014-12-10

**Authors:** Paul Valentich-Scott, Charles L. Powell, II, Thomas D. Lorenson, Brian E. Edwards

**Affiliations:** 1 Santa Barbara Museum of Natural History, 2259 Puesta del Sol Road, Santa Barbara, CA 93105; 2 U.S. Geological Survey, 345 Middlefield Road, Menlo Park, CA 94025

**Keywords:** Thyasiridae, Beaufort Sea, Alaska, Mollusca, Bivalvia, *Maorithyas*, *Wallerconcha*, *Spinaxinus*, *Axinus*, *Parathyasira*, chemoautotrophic, endosymbiosis, taxonomy, Arctic Ocean

## Abstract

Bivalve mollusk shells were collected in 2350 m depth in the Beaufort Sea, Arctic Ocean off northern Alaska. Initial identification suggested the specimens were a member of the bivalve family Thyasiridae, but no known eastern Pacific or Arctic living or fossil thyasirid resembled these deep-water specimens. Comparisons were made with the type of the genera *Maorithyas* Fleming, 1950, *Spinaxinus* Oliver & Holmes, 2006, *Axinus* Sowerby, 1821, and *Parathyasira* Iredale, 1930. We determined the Beaufort Sea species represents a new genus, herein described as *Wallerconcha*. These specimens also represent a new species, herein named *Wallerconcha sarae*. These new taxa are compared with known modern and fossil genera and species of thyasirds.

## Introduction

In an effort to understand the tectonic and sedimentary history of the Arctic Ocean between Canada and Alaska, a joint US-Canadian ice breaker expedition working under the sponsorship of the Extended Continental Shelf Project conducted operations in the Canada Basin during August 2010. The primary mission of the expedition was to collect seismic-reflection and high-resolution bathymetric data. Occasionally there was an opportunity to collect gravity and piston core samples throughout the basin. One of these cores was collected on a mound previously identified on seismic records. Bivalve mollusk specimens were collected in some of these samples and have provided the material for this paper.

## Geologic setting

The informally named “Canning Seafloor Mound” (Hart et al. 2011; hereafter referred to as the Canning Seafloor Mound), a probable cold seep, overlies the crest of a buried anticline in a region of sub-parallel compressional folds beneath the eastern Beaufort Sea outer slope. The collecting locality is adjacent to the frontier oil and gas regions offshore of Prudhoe Bay. The basin is host to extraordinarily deep sedimentary sections about 10 km thick with high organic matter content from river discharge, enhancing the probability of oil and gas generation at depth (Grantz et al. 2011, Grantz and Hart 2012).

## Taxonomic background

Bernard (1972) reviewed the thyasird bivalves in western Canada. He examined specimens from throughout the northeast Pacific as well in the Arctic. In this treatment he synonymized a large number of genera into *Thyasira* Lamark, 1818, including *Axinus* G.B. Sowerby I, 1821 and *Conchocele* Gabb, 1866. Both genera are now known to be distinct (Oliver and Holmes 2007; Coan and Valentich-Scott 2012).

The only systematic treatment that included deep-water Beaufort Sea bivalves was presented by Bernard (1979). In the Beaufort Sea he documented four species of Thyasiridae between the intertidal zone and 2560 m, including a minute deep-water species *Axinulus careyi* Bernard, 1979.

Kristofovich (1936) reported on the *Thyasira* of Tertiary deposits on the western coast of Kamchatka, Russia. Fossil and modern species of *Thyasira* from northeastern Honshu, Japan, were detailed by Yabe and Nomura (1925).

Considerable research has been published in the last 15 years on thyasirds from cold seeps and hot vents (Oliver and Sellanes 2005, Oliver and Holmes 2006, 2007b, Oliver and Levin 2006, Taylor et al. 2007, Zelaya 2009, Oliver et al. 2013, Hryniewicz et al. 2014, Oliver 2014) and their associated with chemosynthetic communities, which are now known to be distributed widely throughout the world’s oceans (Sibuet and Olu 1998, Fujikura et al. 1999, Fujiwara et al. 2001). The Canning Seafloor Mound probably represents a cold seep habitat (Hart et al. 2011).

Here we describe a new genus and new species of thyasirid bivalve from a deep-water seafloor mound in the eastern Beaufort Sea, off northern Alaska and compare it to other thyasirid genera and species.

## Materials, methods, abbreviations

**Figure 1.**
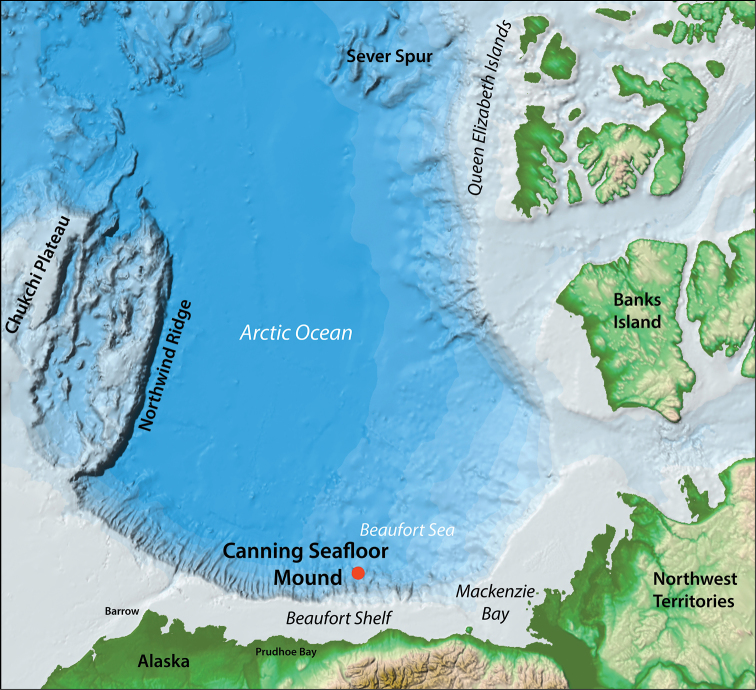
Base map adapted from Jakobsson et al. (2008) showing the location of the Canning Seafloor Mound off the coast of northern Alaska.

**Figure 2.**
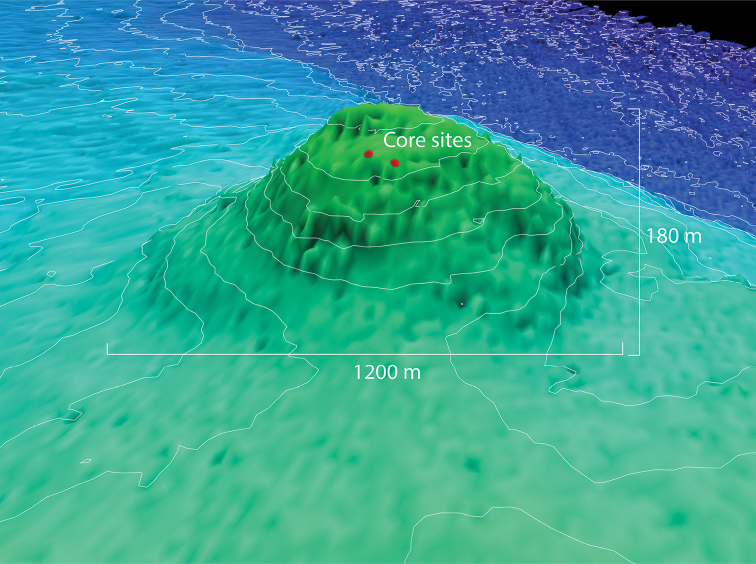
Shaded seafloor bathymetry showing shape and approximate dimension of the Canning seafloor mount. The core sites are shown as red dots.

Bivalve specimens were examined from cores collected by the USCGC *Healy* (cruise HLY1002; USGS Station FAID H–3–10–AR; 71.3176°N, 143.9982°W) from the Canning Seafloor Mound, at a depth of 2530 m in the Beaufort Sea off northern Alaska (Figure 1). The Canning Seafloor Mound is conical and approximately 1200 m diameter and 180 m high (Figure 2).

The bivalve specimens were recovered in two cores (1P–1 and 1GB–1) from 0.02 to 4.65 meters below the seafloor. The greater depth indicates long-term residence of the bivalves (see below under Age), whereas the shallow depth and assumed young age could indicate that this species might still be living on or near the mound. The shells are associated with gas hydrate, methane saturated sediments and authigenic carbonates (Edwards et al. 2011, Hart et al. 2011, Lorenson et al. 2011, Pohlman et al. 2011). This strongly suggests that the bivalves had chemotrophic endosymbionts similar to other bivalves that inhabit active cold vents (Roberts and Carney 1997, Fujiwara et al. 2001, Oliver 2014).

For Figure 7, diagrammatic line drawings were made from digital images of the holotypes of each species. Outlines of each type specimen were made in Adobe Photoshop by selecting all space outside of the shell, inversing the selection and creating a clipping path along the shell edge. With the clipping path selected, we processed the “stroke path” command.

The following abbreviations are used in the text: ECS – Extended Continental Shelf; FAID – field activity identification; GNS – GNS Science, Lower Hutt, New Zealand; LACMIP – Invertebrate Paleontology section, Natural History Museum of Los Angeles County, California, USA; mbsf – meters below seafloor; SBMNH – Santa Barbara Museum of Natural History, California, USA; NHMUK – The Natural History Museum, United Kingdom; NMST – National Museum of Nature and Science, Tokyo, Japan; NMW.Z – National Museum of Wales, Zoology, Cardiff, Wales, UK; USGS – United States Geological Survey; USNM – National Museum of Natural History, Smithsonian Institution, Washington D.C., USA.

## Systematic account

### Family Thyasiridae Dall, 1900

#### Wallerconcha

Taxon classificationAnimaliaLucinoidaThyasiridae

Valentich-Scott & C. L. Powell gen. n.

http://zoobank.org/FD1C36AC-1554-4BBE-AFE6-C8955FA39558

Figures 3A–H , 7B

##### Type species.

**Figure 3.**
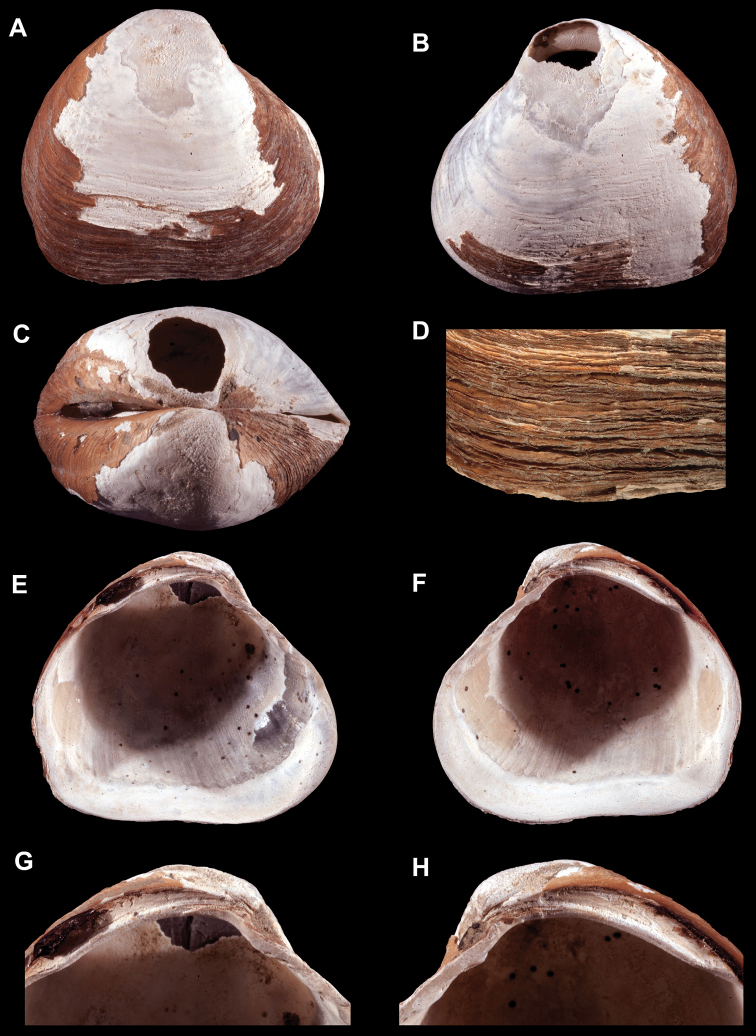
**A–H.** *Wallerconcha sarae* gen. n., sp. n. **A–H** holotype, SBMNH 235481, length = 23.9 mm, height = 21.3 mm, width = 16.7 mm. **A** Exterior of right valve **B** Exterior of left valve **C** Dorsal view of both valves **D** Close up of periostracum of right valve **E** Interior of left valve **F** Interior of right valve **G** Close up of hinge of left valve **H** Close up of hinge of right valve.

*Wallerconcha sarae* Valentich-Scott & C.L. Powell, new species herein (Figures 3A–H, 7B). No other species are currently included in the genus.

##### Description.

Shell moderate in size (length to 24 mm), subtrigonal, subequilateral, strongly inflated; beaks broad, strongly prosogyrous; posterior radial sulcus shallow; sculpture of moderate to strong, uneven commarginal ribs and striae; periostracum thick, dehiscent, medium to dark brown, wrinkled, without micro-spines; lunule absent; escutcheon long, moderately narrow, moderately impressed; ligament large, long, deeply sunken on a stout nymph; hinge edentulous or with minute tubercles; hinge fig well defined and strongly thickened posteriorly; anterior adductor muscle scar wide, long.

##### Etymology.

The genus is named in honor of Thomas R. Waller (Smithsonian Institution) for his significant contributions to our understanding of the evolution, biogeography and systematics of fossil and modern marine bivalves.

##### Comparisons.

*Wallerconcha* differs from all other members of the Thyasiridae by the combination of four primary shell characteristics: 1) a well-defined hinge fig; 2) a heavy, deeply sunken nymph; and 3) a broad, elongate anterior adductor muscle scar that is not divided into two sections; 4) a dark, thick, wrinkled periostracum, without micro-spines.

**Figure 4.**
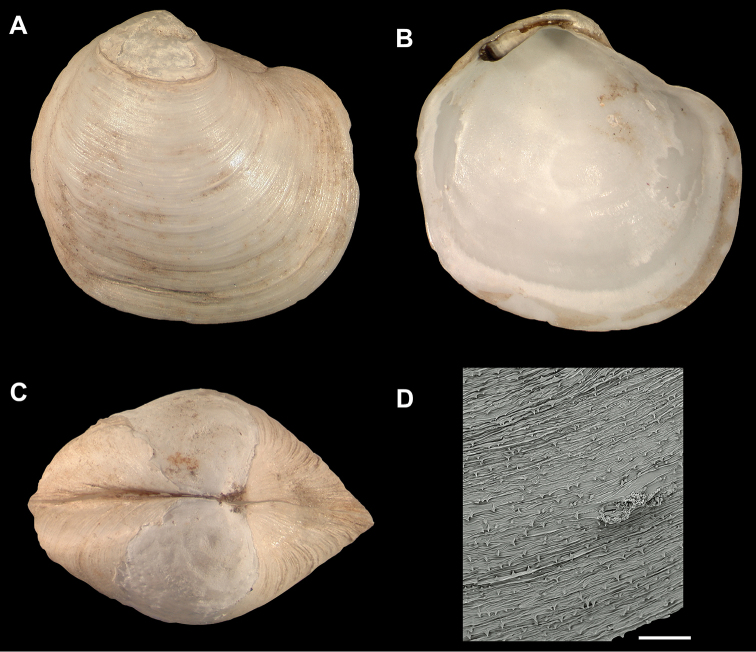
**A–D** *Spinaxinus sentosus*. **A–G** holotype, NMW.Z. 2002.108.1, length = 13.5 mm, height = 13.3 mm, width = 8.6 mm. **A** Exterior of right valve **B** Interior of left valve **C** Dorsal view of both valves **D** Scanning electron micrograph of periostracum, scale bar = 200 µm. Photo credit P. Graham Oliver and Anna M. Holmes, National Museum of Wales.

*Wallerconcha* is similar to the deep-water genus *Spinaxinus* Oliver & Holmes, 2006 (type species, *Spinaxinus sentosus* Oliver & Holmes, 2006) (Figure 4A–D). The latter genus has a thin, translucent, minutely spinose periostracum (Figure 4D), whereas the periostracum of *Wallerconcha* is thick and wrinkled but lacks periostracal spines (Figure 3D). In addition, *Wallerconcha* has a much longer and wider anterior adductor muscle scar, and a longer and deeper nymph.

Another similar genus is *Axinus* G.B. Sowerby I, 1821 (type species *Axinus angulatus* G.B. Sowerby I, 1821). Oliver and Holmes (2007a) reviewed several members of this genus and concluded that it has a large lunule, a moderate to strong posterior radial sulcus, a thin hinge fig, and lacks a heavy nymph, all features which separate it from *Wallerconcha*.

*Parathyasira* Iredale, 1930 (type species *Parathyasira resupina* Iredale, 1930) has an external sculpture of minute rows of spines, and a distinct radial sulcus. It also has a thin hinge fig and weak nymph, which are less robust than *Wallerconcha*. Both genera have an elongate anterior adductor muscle scar, whereas in *Parathyasira* the scar is usually divided into several sections, *Wallerconcha* has a single, broad scar.

**Figure 5.**
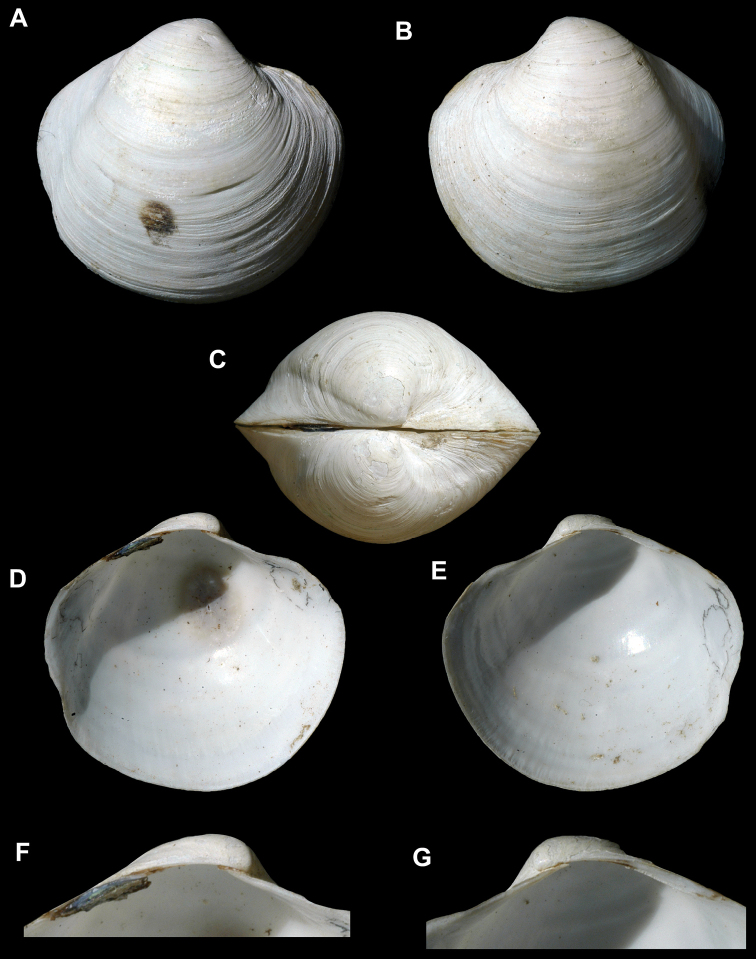
**A–G** *Maorithyas marama*, holotype, GNS–TM 305, length = 18.7 mm, height = 17.2 mm, width = 13.7 mm. **A** Exterior of right valve **B** Exterior of left valve **C** Dorsal view of both valves **D** Interior of left valve **B** Interior of right valve **F** Close up of hinge of left valve **G** Close up of hinge of right valve.

*Maorithyas marama* Fleming, 1950, the type species of the genus, has a very thin hinge fig, lacks a heavy nymph, and has a shorter anterior adductor muscle scar (Figures 5A–G, 7A) when compared to *Wallerconcha*.

**Figure 6.**
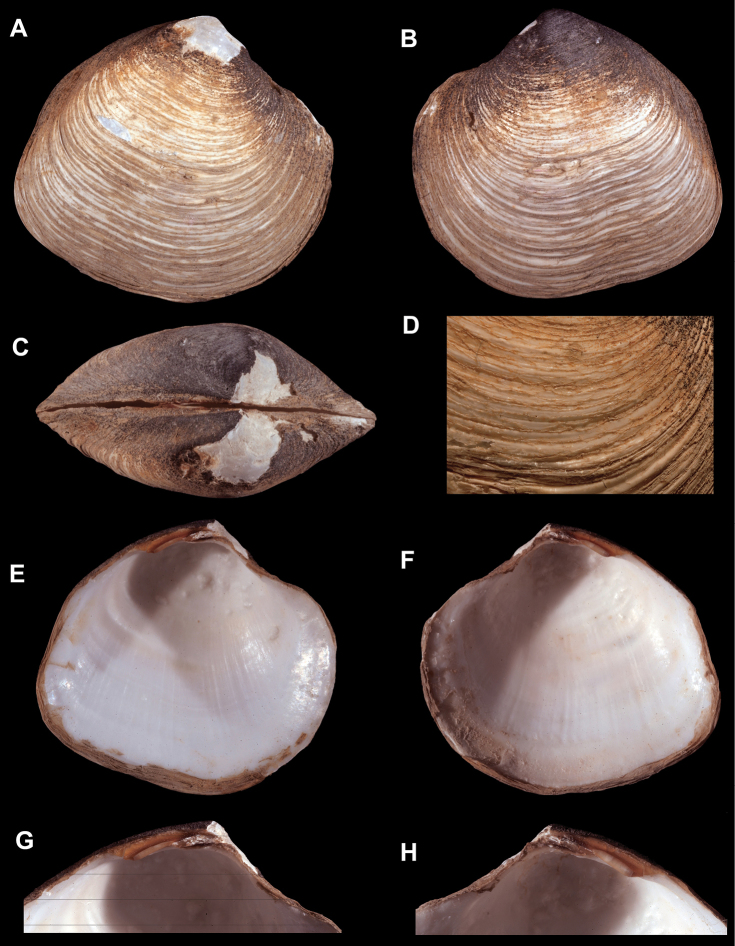
**A–H** *Maorithyas hadalis*, holotype, NSMT 71431, length = 26.7 mm, height = 24.1 mm, width = 13.4 mm. **A** Exterior of right valve **B** Exterior of left valve **C** Dorsal view of both valves **D** Close up of periostracum of right valve **E** Interior of left valve **F** Interior of right valve **G** Close up of hinge of left valve **H** Close up of hinge of right valve.

Okutani et al. (1999) placed their new, deep-water Japanese thyasirid species into the shallow-water genus *Maorithyas* Fleming, 1950. They chose the generic placement of *Maorithyas hadalis* Okutani et al., 1999 based on the shallow posterior radial sulcus, and relatively heavy sculpture. The internal shell characteristics of *Maorithyas hadalis* (holotype, NSMT 71431), namely the periostracum, hinge fig, nymph and anterior adductor muscle scar place it outside of *Maorithyas* or *Wallerconcha* (Figure 6A–H). It potentially belongs in a new genus, but that description is outside the scope of this paper.

**Figure 7.**
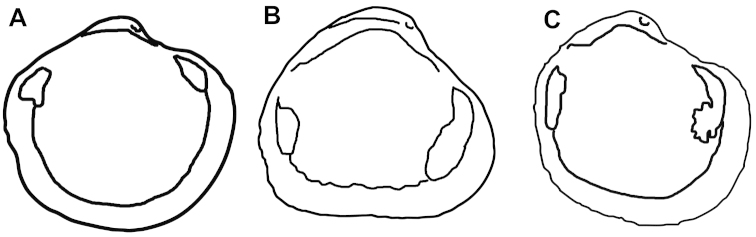
Comparison of adductor muscle scars and pallial lines of left valves of holotypes. **A** *Maorithyas marama*, holotype **B** *Wallerconcha sarae*, holotype **C** *Spinaxinus sentosus*, holotype. – Not to scale.

#### Wallerconcha sarae

Taxon classificationAnimaliaLucinoidaThyasiridae

Valentich-Scott & C.L. Powell sp. n.

http://zoobank.org/70B6274D-A766-48E3-B33E-4D353E78F69D

Figure 3A–H , 7B

##### Description.

Shell shape. Shell subtrigonal, moderately thin, equivalved, highly inflated; anterior margin broadly rounded; posterior end subtruncate; umbo broadly rounded, strongly prosogyrate; dorsal margin strongly sloping on both sides of the umbo; escutcheon moderately narrow, moderately deep, well-defined; lunule absent. Maximum length 24 mm, maximum height 24 mm, maximum width 17 mm.

Sculpture and periostracum. Shell with closely spaced, irregular commarginal striae and ribs; shallow, narrow radial sulcus extends from posterior of the umbo to the posterior ventral margin; shallow radial depression from the umbo to the central ventral margin, forming a slight undulation along the ventral margin; periostracum thick, wrinkled, dehiscent, light to dark brown, silky.

Hinge. Hinge heavy, edentulous, or with minute tubercles under beaks; anterior section narrow; posterior section with wide lateral platform, supporting deeply sunken nymph; ligament external, deeply sunken, long, dark brown.

Adductor muscle and pallial scars – anterior adductor muscle scar large, long, wide, subelliptical, with irregular upper and lower margins, upper margin of scar concave near the center; posterior adductor muscle scar smaller, irregular ovate, with a pointed projection in juveniles; pallial line scalloped, without a sinus.

Interior – interior dirty white to gray; with faint radial crescent-shaped lines that extend from near the umbo to the near the ventral margin, lines have broad depressions between them near the central ventral margin.

##### Type locality.

USA, Alaska, Beaufort Sea, Canning Seafloor Mound. Specifically, 71.317°N, 143.999°W; 2,358 m water depth (ECS004 137, Core IP–1, section 3, 31 cm, 4.65 mbsf).

##### Type specimens.

Holotype – SBMNH 235481, 1 pair, length = 23.9 mm, height = 21.3 mm, width = 16.7 mm. Alaska, Beaufort Sea, Canning Seafloor Mound; 71.317°N, 143.999°W; 2,358 m water depth (ECS004137, Core IP–1, section 3, 31 cm; 4.65 mbsf)

Paratype 1 – CAS paratype 72852

Alaska, Beaufort Sea, Canning Seafloor Mound; 71.317°N, 143.998°W; 2,350 m water depth (ECS 004 122. Core 1GB–1 102 cm, 1.02 mbsf); length = 12.8 mm, height = 10.9 mm

Paratype 2 – LACMIP paratype 14470

Alaska, Beaufort Sea, Canning Seafloor Mound; 71.317°N, 143.999°W; 2,358 m water depth (ECS004242, Core IP1, section 1, 52 cm, 2/2, 0.52 mbsf); length = 15.0 mm, height = 13.1 mm

Paratype 3 – SBMNH paratype 235613

Alaska, Beaufort Sea, Canning Seafloor Mound; 71.317°N, 143.998°W; 2,350 m water depth (ESC004180, Core 1GB–1, 44 cm, 0.44 mbsf); length = 19.2 mm, height = 17.5 mm

Paratype 4 – SBMNH paratype 235614

Alaska, Beaufort Sea, Canning Seafloor Mound; 71.317°N, 143.998°W; 2,350 m water depth (ESC004180, Core 1GB–1, 44 cm, 0.44 mbsf); length = 23.9 mm, height = 23.8 mm.

##### Etymology.

Named in honor of Sara Powell, of San Jose, California, daughter of Charles L. Powell.

##### Distribution.

*Wallerconcha sarae* is presently only known only from the region around the type locality; the Canning Seafloor Mound (71.3175°N, 143.9997°W), Beaufort Sea, Alaska, USA. Given the collection depth of 0.02–4.65 mbsf, we surmise this is a fossil species. However we cannot discount that it could still be living in the region.

##### Other specimens examined.

Piston core: ESC 004112, Core 1P1, section 4, 15 cm, 4.65 mbsf (articulated specimen; frozen for further analysis), ECS 004137, Core 1P1, section 3, 31 cm, 181 mbsf (one articulate specimen (holotype Figure 3A–G), three larger fragments), ESC 004242, 1P1, sec. 1, 52 cm, 0.52 mbsf, (one left valve), ESC 004242, 1P1, sec. 1, 52–54 cm, 0.53 mbsf (seven large fragments), ECS 004242, 1P1, sec. 1, 52–54 cm, 0.53 mbsf (one fragment). Gravity Core: ESC 004115, Core 1GB1, 0.02 mbsf (left valve; used for chemical analysis), ESC 004122, 1GB1, 102 cm, 1.02 mbsf (one small left valve), CS 004180, Core 1GB1, 44 cm, 0.44 mbsf (one articulate specimen, one left valve, two fragments). EESC 004257, Core 1TC1, section 1, 72 cm, 0.72mbsf (two larger fragments).

## Comparisons

The new species has shell characteristics closest to “Maorithyas” hadalis Okutani et al., 1999 (Figures 6A–H), collected from over 7,000 m in the Japanese Trench. *Wallerconcha sarae* is much more inflated, has broader umbones, and a much longer ligament and nymph. When compared to *Maorithyas hadalis*, *Wallerconcha sarae* has a much larger, broader, and more elongate anterior adductor muscle scar.

There are also similarities between *Wallerconcha sarae* and members of the genus *Spinaxinus* Oliver & Holmes, 2006. However, all of the currently described species in this genus have a minutely spinose periostracum. The eastern Atlantic *Spinaxinus sentosus* (Figures 4A–D) is less inflated than *Wallerconcha sarae*, has narrower beaks, and a smaller anterior adductor muscle scar (Figure 7C). *Spinaxinus emicatus* Oliver in Oliver et al., 2013, from the Gulf of Mexico is compressed and circular in outline, has narrow beaks, an evident radial sulcus, and a much shorter nymph when compared to *Wallerconcha sarae*. The Fijian *Spinaxinus phrixicus* Oliver in Oliver et al., 2013, is also compressed and circular in outline with narrow beaks, but it has a distinctive shell sculpture of commarginal ridges.

The minute deep-water Beaufort Sea thyasirid, *Axinulus careyi* is much smaller (maximum length 2.7 mm), has a more defined escutcheon, and lacks the broad posterior hinge fig. It also has a relatively short, narrow anterior adductor muscle scar when compared to the long broad scar of *Wallerconcha sarae*.

*Axinus grandis* (Verrill & Smith in Verrill, 1885) and *Axinus cascadiensis* Oliver & Holmes, 2007 have a few external similarities to *Wallerconcha sarae*. *Axinus grandis* is an Atlantic and Mediterranean species, that is easily separated from *Wallerconcha sarae* by its roughly diamond-shaped shell outline. *Axinus cascadiensis* is known only from a seamount off Oregon (Oliver and Holmes 2007) and the shell outline serves to separate *Axinus cascadiensis* from *Wallerconcha sarae*. With *Axinus cascadiensis* being less inflated, having narrower, more prosogyrate umbos, and a strong anterior protrusion. In addition, the escutcheon of *Axinus cascadiensis* is larger and more deeply impressed.

The Cretaceous fossil *Thyasira becca cobbani* Kauffman, 1967 (pl. 5, f. 34, 35; 1969, pl. 127, f. 20) has a deep radial sulcus and narrow, strongly prosogyrate beaks. *Thyasira becca cobbani* is known from the western interior of the North America in the Pierre Shale, Upper Cretaceous (Campanian-Maastrichtian) of Pueblo County, Colorado and in the Riding Mountain Formation, Upper Cretaceous (Campanian-Maastrichtian) exposed along the Assiniboine River, Manitoba, Canada. *Thyasira alaskensis* Kauffman, 1969, described from the Miocene and (or) Pliocene Nuwok Formation Member of the Sagavanirktok Formation on the Alaskan North Slope is easily separated by its more rounded outline, smaller and less prosogyrate umbo, and in having a prominent sulcus, although it is reportedly closely related to *Thyasira becca cobbani* (Kauffman, 1967). Both of these fossil species have narrow hinge fig, narrow, strongly prosogyrate beaks and a deep radial sulcus, all of which excludes them from *Wallerconcha*.

### Age

The sedimentation rate in this region, derived from seismic lines in Grantz et al. (2011) showing the depth of the Quaternary section at this approximate location, is estimated to be about 0.5 m per 1000 years. Measured sedimentation rates upslope of our site on the nearby Mackenzie prodelta by Bringué and Rochon (2012) of 1.43 m/1000 years indicates our estimated rates are reasonable. The sedimentation rate suggests that *Wallerconcha sarae* has been continuously present here from about 10,300 years to the near present. The age estimate is derived from the interspersed presence of the *Wallerconcha sarae* specimens from 0.02–5.16 mbsf in our suite of cores, where 5.16 m of sediment corresponds to an accumulation time of 10,320 years. The actual maximum age is likely greater because we have not taken sediment compaction into account, and there is a distinct possibility that *Wallerconcha sarae* is present below the penetration depth of our core samples.

Although we cannot be certain that *Wallerconcha sarae* is extinct, we have used associated specimens to determine the potential age of the deposits where it was collected. The planktic foraminiferan *Neogloboquadrina pachyderma* (Ehrenberg 1861), a species that has been extinct for 1.8 million years, was collected from the base of the same cores as *Wallerconcha sarae* at the same depth as the holotype specimen (4.65 mbsf), thus indicating an early Pleistocene age (Wan et al. 2011). A gastropod columella and part of the upper spire of *Neptunea* (Mollusca: Gastropoda: Buccinidae) was found at the Canning Seafloor Mound (ECS004230, Core 1P1, section 2, 31 cm) and associated with *Wallerconcha sarae*. *Neptunea* are predatory snails well represented in the earliest Miocene to Holocene of the northern Pacific and in the late Pliocene to Holocene of the Arctic and northern Atlantic. The presence of *Neptunea* gives a maximum age for these deposits of latest Miocene or early Pliocene, after the opening of the Bering Strait (Marincovich and Gladenkov 1999; Marincovich et al. 2002).

## Supplementary Material

XML Treatment for Wallerconcha

XML Treatment for Wallerconcha sarae
